# 1598. Predictive equations for immune recovery at 48 weeks of BIC/FTC/TAF treatment in Virologically Suppressed PLWHIV.

**DOI:** 10.1093/ofid/ofad500.1433

**Published:** 2023-11-27

**Authors:** Roberto Pedrero Tomé, Luis Buzón, Guillermo Pousada, Carlos Dueñas, Carlos Galera, Jose Sanz, Jesús Troya

**Affiliations:** Hospital Universitario Infanta Leonor, Madrid, Madrid, Spain; Hospital de Burgos, Burgos, Castilla y Leon, Spain; Hospital Àlvaro Cunqueiro, Vigo, Galicia, Spain; Hospital de Valladolid, Valladolid, Castilla y Leon, Spain; H. L´Arrixaca Murcia, Murcia, Murcia, Spain; Hospital Principe de Asturias, Alcalá de Henares, Madrid, Spain; Hospital Universitario Infanta Leonor, Madrid, Madrid, Spain

## Abstract

**Background:**

The possibility to anticipate immune recovery could be of great interest when a switching strategy is planned, especially among those patients with low CD4+ count as an indirect marker of potential immunological success not only in absolute CD4+ and CD8+ count but also with CD4/CD8 ratio. The objective is to provide predictive equations that allow prognostication of 48-week immune recovery in HIV-positive men and women treated with BIC/FTC/TAF based on baseline CD4+ and CD8+ cell counts.

**Methods:**

We collected data from 1315 adult patients with undetectable HIV+ who switched to a bictegravir/emtricitabine/tenofovir triple therapy regimen (1028 men; 287 women). We studied CD4+ and CD8+ cell counts at baseline and 48 weeks. Simple linear regression models were performed to predict patient immune status at 48 weeks according to patient sex. A logarithmic transformation of each variable was performed to obtain equations with higher predictive power. Each simple linear regression model was validated by an ANOVA test (p < 0.001). In addition, the assumptions of linearity, normality of residuals (Shapiro-Wilk test), homoscedasticity (Breusch-Pagan test), and independence of residuals were verified using the Durbin-Watson test. Each model was internally validated with one-third of the dataset using a bootstrap of 5000 iterations.

**Results:**

All four proposed equations yield good predictive values (adjusted R-squared: 0.705 - 0.835). In the case of women, the proposed formulae are as follows: [1] 48 weeks Log10 [CD8+ cells/mm^3^] = 0.923 + 0.671*Baseline Log10 [CD8+ cells/mm3] (adjusted R-squared = 0.835); [2] 48 weeks Log10 [CD4+ cells/mm3] = 0.702 + 0.757*Baseline Log10 [CD4+ cells/mm^3^]] (adjusted R-squared = 0.746). For males, the formulas are as follows: [1] 48 weeks Log10 [CD8+ cells/mm^3^] = 1.392 + 0.571*Baseline Log10 [CD8+ cells/mm^3^] (adjusted R-squared = 0.705); [2] 48 weeks Log10 [CD4+ cells/mm^3^] = 0.605 + 0.795*Baseline Log10 [CD4+ cells/mm^3^] (adjusted R-squared = 0.810). The results obtained in the bootstrap do not differ from the initial models and therefore support the equations proposed.

**Conclusion:**

Available data on immunological recovery in patients with triple therapy are scarce, and this could be an adjunct to predicting the evolution of these patients.

**Disclosures:**

**Luis Buzón, MD, PhD**, Janssen: Advisor/Consultant|Janssen: Expert Testimony|MSD: Expert Testimony|Viiv: Advisor/Consultant|Viiv: Expert Testimony **Carlos Dueñas, MD**, Gilead: Advisor/Consultant|Gilead: Expert Testimony|Jannsen: Advisor/Consultant|Jannsen: Expert Testimony **Jose Sanz, MD, PhD**, Gilead: Expert Testimony|Janssen: Expert Testimony|MSD: Expert Testimony|ViiV: Expert Testimony **Jesús Troya, Staff**, Gilead: Advisor/Consultant|Gilead: Expert Testimony|Janssen: Board Member|Janssen: Grant/Research Support|ViiV: Advisor/Consultant

